# BTG1 inhibits malignancy as a novel prognosis signature in endometrial carcinoma

**DOI:** 10.1186/s12935-020-01591-3

**Published:** 2020-10-07

**Authors:** Yibing Li, Jianing Huo, Junjian He, Yunzheng Zhang, Xiaoxin Ma

**Affiliations:** Department of Obstetrics and Gynecology, Shengjing Hospital of China Medical University, 39 Huaxiang Road, Tiexi District, Shenyang, 110000 Liaoning People’s Republic of China

**Keywords:** Endometrial carcinoma, BTG1, Bioinformatics, Prognosis, EMT, Proliferation, Migration, Invasion, Apoptosis

## Abstract

**Background:**

Endometrial carcinoma (EC) is one of the three major malignant tumors of the female reproductive system. In recent years, the incidence and mortality rate of EC have increased. B-cell translocation gene 1 (BTG1) is an anti-proliferation gene that regulates the occurrence and development of a variety of tumors, but there is no research regarding this gene in EC.

**Methods:**

Based on The Cancer Genome Atlas (TCGA) database, we used a variety of bioinformatics tools and databases to explore the expression and prognosis of BTG1. We verified expression and prognosis of BTG1 in EC using qRT-PCR and analyzed the relevant clinicopathological parameters. We functionally enriched BTG1 and related genes in EC patients through the bioinformatics website and analyzed miRNA targets of BTG1 and interacting protein networks. Cell proliferation, wound healing, transwell invasion, and cell apoptosis assays were used to detect the effects of BTG1 on the malignant biological behavior of endometrial carcinoma cells (ECCs). The effect of BTG1 on the epithelial-to-mesenchymal transition (EMT) process was detected using western blot.

**Results:**

We analyzed the expression and prognosis of BTG1 based on TCGA and found that low expression of BTG1 was associated with poor EC prognosis. The qRT-PCR suggested that BTG1 had low expression in EC. BTG1 expression was significantly correlated with overall survival (OS) shortening. Clinicopathological analysis suggested that expression of BTG1 was related to invasion depth and the International Federation of Gynecology and Obstetrics (FIGO) stage. EC pathological tissue type, fertility history, lymphatic metastasis, menopause, estrogen receptor (ER), progesterone receptor (PR), and age of diagnosis were not related. Functional enrichment analysis showed that BTG1 plays an important role in regulating embryonic development, tumorigenesis, apoptosis, and cell cycle. Biological behavior experiments suggest that BTG1 inhibits proliferation, migration, and invasion of ECCs, and promotes apoptosis of ECCs. Western blot indicated that BTG1 inhibited the EMT process of ECCs.

**Conclusions:**

BTG1, as a tumor suppressor gene, plays an important role in the occurrence and development of EC. We believe that BTG1 can be used as a potential prognostic biomarker for EC.

## Background

Endometrial carcinoma (EC) is the sixth most common cancer among women worldwide. It is also one of the most common tumors of the female reproductive system [1]. Recently, the incidence and mortality rate of EC has increased [2]. The risk factors for EC include elevated estrogen levels (caused by obesity, diabetes, and high-fat diets), premature menarche, being non-parturients, late desperate age, Lynch syndrome, grade ≥ 55 years old, and using tamoxifen [3]. The mortality rate of EC is directly related to poor prognostic factors driving tumor recurrence [4].

B-cell translocation gene 1 (BTG1) is a member of the TOB/BTG protein family and is an anti-proliferation gene. Studies have shown that the protein encoded by the TOB/BTG protein family inhibits cell proliferation, induces cell apoptosis and cell cycle arrest, and regulate the development of many cell types [5–7]. It also prevent tumor neovascularization and the expression of vascular endothelial growth factor [8]. The protein product of BTG1 is involved in various cellular processes such as cell division, DNA repair, transcriptional regulation, and messenger RNA stability. BTG1 can also affect the developmental process, adult differentiation, and homeostasis regulation under conditions of cell stress [9]. Multiple studies have found associations between BTG1 and various tumors. It has been shown to be abnormally expressed in gastric cancer, kidney cancer, hepatocellular carcinoma, thyroid carcinoma, nasopharyngeal carcinoma, ovarian cancer, breast cancer, non-small cell lung cancer, glioblastoma and cervical cancer [10–19].

However, the relationship of BTG1 with EC has not been studied. In this study, we used multiple bioinformatics databases to objectively analyze the expression of BTG1 in EC, its impact on EC prognosis, and analyze the expression of BTG1 in the function regulation network. In addition, we studied the expression of BTG1 in EC tissues and its relationship with clinical pathological parameters and prognosis. Through in vitro studies we explored the effect of BTG1 on the malignant biological behavior of endometrial cancer cells (ECCs) and epithelial-to-mesenchymal transition (EMT).

## Materials and methods

### Oncomine database analysis

This study used the Oncomine database (http://www.oncomine.org) [20]. It can be used to analyze gene expression differences, find outliers, and predict co-expression. Through this database, we analyzed the expression of BTG1. The cut-off setting was set to P value < 0.05.

### GEPIA database analysis

GEPIA (http://gepia.cancer-pku.cn) [21] is a website based on TGCA and GTEx projects cancer data mining. We analyzed the differential expression of BTG1 in tumors and normal tissues through GEPIA. Gene name: BTG1.

### UALCAN database analysis

UALCAN (http://ualcan.path.uab.edu) [22] is a TCGA database online analysis and mining website, built on PERL-CGI, javascript, and css. Based on this database, we analyzed the relative expression of BTG1 in EC tissue and normal endometrial tissue. We analyzed the relative expression in different subgroups of EC, including: individual cancer stages, patient’s weight, patient’s age, histological subtypes, TP53 mutation status, patient’s race, and menopause status. In addition, UALCAN was used to analyze the prognosis of EC.

### Survival analysis

Kaplan–Meier (KM) plotter (http://kmplot.com) [23] is an online tool for analyzing the prognosis of tumor patients. According to the expression of BTG1, EC patients were divided into a BTG1 high-expression group and a BTG1 low-expression group. The follow-up time was set at 120 months.

### LinkedOmics database analysis

In this study, the LinkFinder module of the LinkedOmics database (http://www.linkedomics.org/login.php) [24] was used to study differentially expressed genes related to BTG1 with nTM = 176 in the TCGA UCEC dataset. The Pearson correlation coefficient was used to statistically analyze the results. LinkFinder displays the results in the form of volcano and heat maps.

### Metascape analysis

Metascape (http://metascape.org) [25] is a powerful gene function annotation analysis tool. We used Metascape to analyze the enrichment of the top 500 genes of BTG1, and its related differential expression through processes and pathways. Among them, P ≤ 0.01, the minimum count was 3, and the enrichment factor was > 1.5, to obtain significant statistical differences.

### GeneMANIA analysis

GeneMANIA (http://www.genemania.org) [26] can predict gene function and analyze gene lists to look for interaction networks between genes, relationships between genes, and also analyze protein–protein, protein–DNA interactions, pathways, physiological and biochemical reactions, co-expression, and co-localization. Through GeneMANIA, we obtained a network of interactions between genes with significant differences in BTG1.

### miRDB analysis

miRDB (http://mirdb.org) [27] is an online database for miRNA target prediction and functional annotation. All targets in miRDB were predicted by the bioinformatics tool MirTarget. We used miRDB to find all miRNAs that bind to BTG1.

### Human tissue specimens

All EC tissue samples and normal endometrial tissue samples were from patients undergoing total hysterectomy at Shengjing Hospital affiliated to China Medical University. All patients had normal thyroid hormones (TSH and FT4), prolactin, fasting blood sugar, Liver tests, Blood urea nitrogen, creatinine, Infectious tests, coagulation factors and serum levels of sodium, potassium. These patients were all between 30 and 80 years old and had not used any drugs for 1 month before the operation. In the 70 patients with EC included in this study, all patients had no history of other malignancies. The diagnosis of EC was evaluated by two experienced clinical pathologists based on FIGO for histological diagnosis and tumor grading. None of the patients received chemotherapy, radiotherapy and hormones and treatment before surgery. All patients received informed consent, and were approved by the Ethics Committee of Shengjing Hospital Affiliated to China Medical University.

### Extraction of RNA, reverse transcription, and quantitative real-time polymerase chain reaction (qRT-PCR)

Total RNA was extracted from tissues and cells by TRIzol reagent (Takara, Beijing, China). The complementary DNAs (cDNAs) for the lncRNAs and mRNAs of interest were reverse-transcribed from 2 μg of total RNA using PrimeScript RT-polymerase (Takara). qRT-PCR was performed using SYBR-Green Premix (Takara) and specific PCR primers (Sangon Biotech, Shanghai, China). Glyceraldehyde-3-phosphate dehydrogenase (GAPDH) was used as an internal control. The mRNA expression was observed by calculating 2^−ΔΔCT^. The primer sequences are listed in the Additional file 1: Table S1.

### Cell lines, cell culture and transfection

Ishikawa cell line (Shanghai Huiying, Shanghai, China) and HEC-1A cell line (Genechem, Shanghai, China) were cultured in α-MEM medium (Bioind, Kibbutz Beit Haemek, Israel) and McCoy’s 5A medium (Bioind), respectively. The medium contained 10% fetal bovine serum (FBS) (Bioind) and 1% penicillin–streptomycin (Invitrogen). All cells were cultured in a humidified incubator at 37 °C with 5% CO_2_. BTG1’s overexpression plasmid and knockdown plasmid were purchased from GeneChem (Shanghai, China), and both were transfected with jetPRIME^®^ in vitro DNA and siRNA Transfection Reagent (PolyPlus-transfection, France) for subsequent experiments. The related sequences can be found in Additional file 2: Table S2.

### Cell proliferation assay

Cell proliferation assay were performed according our previously described, EdU cell proliferation assay kit (Ribobio, Guangzhou, China) was used to detect the effect of BTG1 on cell proliferation [28]. In a 96-well plate (Guangzhou Jet Bio-Filtration Co., Ltd. Guangzhou, China), 8000 cells were seeded per well. Cellular DNA replication activity was detected by fluorescence microscopy.

### Wound healing assay

Wound healing assay were performed according our previously described [29]. Cells were observed by microscopy and photographed (0 h and 24 h cell scratches). The scratch area was calculated to obtain the migration percentage.

### Transwell invasion assay

Transwell invasion assay were performed according our previously described [28]. Cells were imaged under an inverted fluorescence microscope with an image acquisition system (Nikon, Japan).

### Cell apoptosis assay

After the cells were transfected, 10^6^ cells were collected from each group. After washing once in PBS, the cells were stained with PE Annexin V Apoptosis Detection (BD Pharmingen™, New Jersey, USA) for 15 min at room temperature, and analyzed by flow cytometry (BD FACSCalibur, New Jersey, USA) to assess the proportion of apoptotic cells.

### Western blot

After the cells were harvested, proteins were extracted with the protein extraction kit (Beyotime Biotechnology, Shanghai, China). The samples were separated by 8% sodium dodecyl sulfate–polyacrylamide gel electrophoresis, and then transferred to poly-vinylidene difluoride membranes (Millipore, Massachusetts, USA). The membrane was incubated overnight at 4 °C in diluted primary antibodies against E-cadherin (ProteinTech Group, Chicago, USA), N-cadherin (ProteinTech Group), Vimentin (ProteinTech Group, Chicago, USA). The membrane was then visualized using Quantum One imaging software (Bio-Rad, California, USA). GAPDH was used as an intranuclear control.

### Statistical analysis

The data are expressed as mean ± SEM. All statistical analyses were performed using GraphPad Prism 8.0 Software (La Jolla, CA, USA) and SPSS version 22.0 software (Abbott Laboratories, Chicago, IL, USA) through the two-sided Student’s t-test or one-way analysis of variance (ANOVA). When P < 0.05, the difference was considered statistically significant. *, *** and *** represent P < 0.05, P < 0.01, and P < 0.001, respectively.

## Results

### Using Oncomine database, GEPIA, and UALCAN to analyze the expression of BTG1

**Fig. 1:**
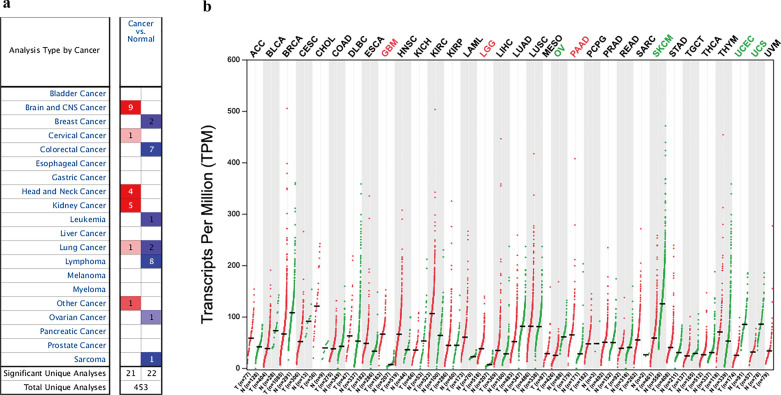
BTG1 expression in cancer and normal tissues. **a** Differential expression of BTG1 in cancer and corresponding normal tissues in Oncomine. **b** The gene expression profile across all tumor samples and paired normal tissues in GEPIA. (Dot plot)

We obtained data from the BTG1 study in 453 different types of tumors through the Oncomine database. A total of 43 studies suggested statistical differences in BTG1 mRNA levels between tumors and normal tissues, of which 21 studies showed that BTG1 expression levels in tumors were significantly increased, and another 22 studies showed BTG1 expression levels in tumors significantly reduced. Compared with normal tissues, BTG1 expression was increased in brain and CNS cancer, cervical cancer, head and neck cancer, kidney cancer, and other cancers. BTG1 showed low expression in breast cancer, colorectal cancer, leukemia, lymphoma, ovarian cancer, and sarcoma. In addition, the expression of BTG1 in lung cancer was increased in one study and decreased in two studies (Fig. 1a). Through GEPIA, we obtained the relative expression of BTG1 in each tumor and normal tissues (Fig. 1b, Additional file 3: Figure S1A).

**Fig. 2:**
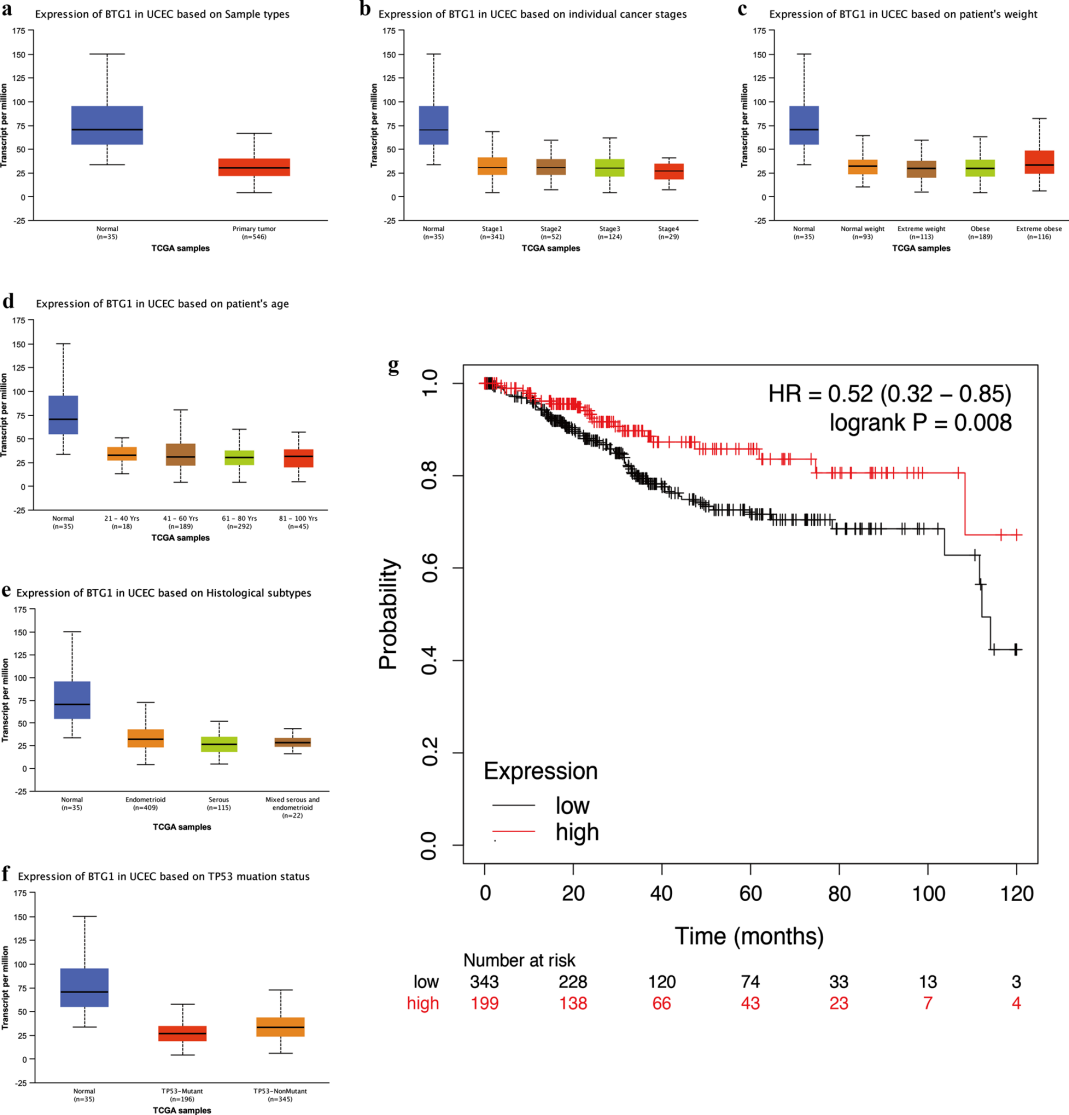
The relationship between BTG1 and various clinical pathological parameters and prognosis in EC. **a** Expression of BTG1 in UCEC based on Sample types in UALCAN. **b** Expression of BTG1 in UCEC based on individual cancer stages in UALCAN. **c** Expression of BTG1 in UCEC based on patient’s weight in UALCAN. **d** Expression of BTG1 in UCEC based on patient’s age in UALCAN. **e** Expression of BTG1 in UCEC based on Histological subtypes in UALCAN. **f** Expression of BTG1 in UCEC based on TP53 mutation status in UALCAN. **g** The effect of BTG1 on the prognosis of endometrial cancer in KM plotter online analysis

Next, we conducted a subgroup analysis of UCEC cases through UALCAN, which included 546 UCEC cases. The results showed that, compared with the normal control group, BTG1 mRNA expression was low in primary endometrial cancer tissues (P = 1.91589999420927E−09; Fig. 2a). The expression level of BTG1 in stage 4 endometrial carcinoma was significantly lower than that in stage 1, stage 2, stage 3 (stage 1 vs stage 4, P = 3.029100E−04; stage 2 vs stage 4, P = 2.382500E−03; stage 3 vs stage 4, P = 4.759200E−03; Fig. 2b). For patients of different weights, there was no difference between normal weight, extreme weight, and obese; however, BTG1 expression of extreme obese group is higher than extreme weight or obese (extreme weight vs extreme obese, P = 8.015500E−03; obese vs extreme obese, P = 2.182600E−03; Fig. 2c). For BTG1 expression in different age groups, the expression level of BTG1 in patients between 61 and 80 years old was lower than the expression level of BTG1 in patients between 41 and 60 years old (age (41–60 years)-vs-Agec(61–80 years), P = 4.821900E−02; Fig. 2d). For the expression level of BTG1 in different histological subtypes, the expression level of BTG1 in endometrioid was higher than in serous and mixed serous and endometrioid subtypes, but there was no difference in expression between serous and mixed serous or endometrioid. (endometrioid vs serous, P = 1.76350000047343E−07; endometrioid vs mixed serous and endometrioid, P = 1.958680E−03; Figure E) Furthermore, the expression level of BTG1 in TP53-Mutant EC was significantly lower than that of TP53-NonMutant EC (TP53-Mutant-vs-TP53-NonMutant; P = 1.71269998183732E−09; Figure F). There was no difference in the expression of BTG1 between patients of different races or menopause status (Additional file 3: Figure S1B-1C).

### High expression of BTG1 can improve EC prognosis

In the KM plotter online analysis tool, we set the follow-up time to 120 months. A total of 543 EC cases in the database met these criteria. The results showed that patients with high BTG1 EC had significantly higher OS than patients with low BTG1 EC (HR = 0.52, 0.32–0.85, logrank P = 0.008) (Fig. 2g). At the same time, in UALCAN, patients with high BTG1 expression (136 patients) had a longer survival time (P = 0.031) compared with patients with low/moderate BTG1 expression (407 patients) (Additional file 3: Figure S1D). However, survival analysis of patients with different menopause status, body weight, and race indicated that there was no difference in survival time (Additional file 3: Figure S1E−1G). These results show that patients of EC with high BTG1 expression have a better prognosis.

### BTG1 mRNA expression in EC and relative clinicopathological analysis

**Fig. 3:**
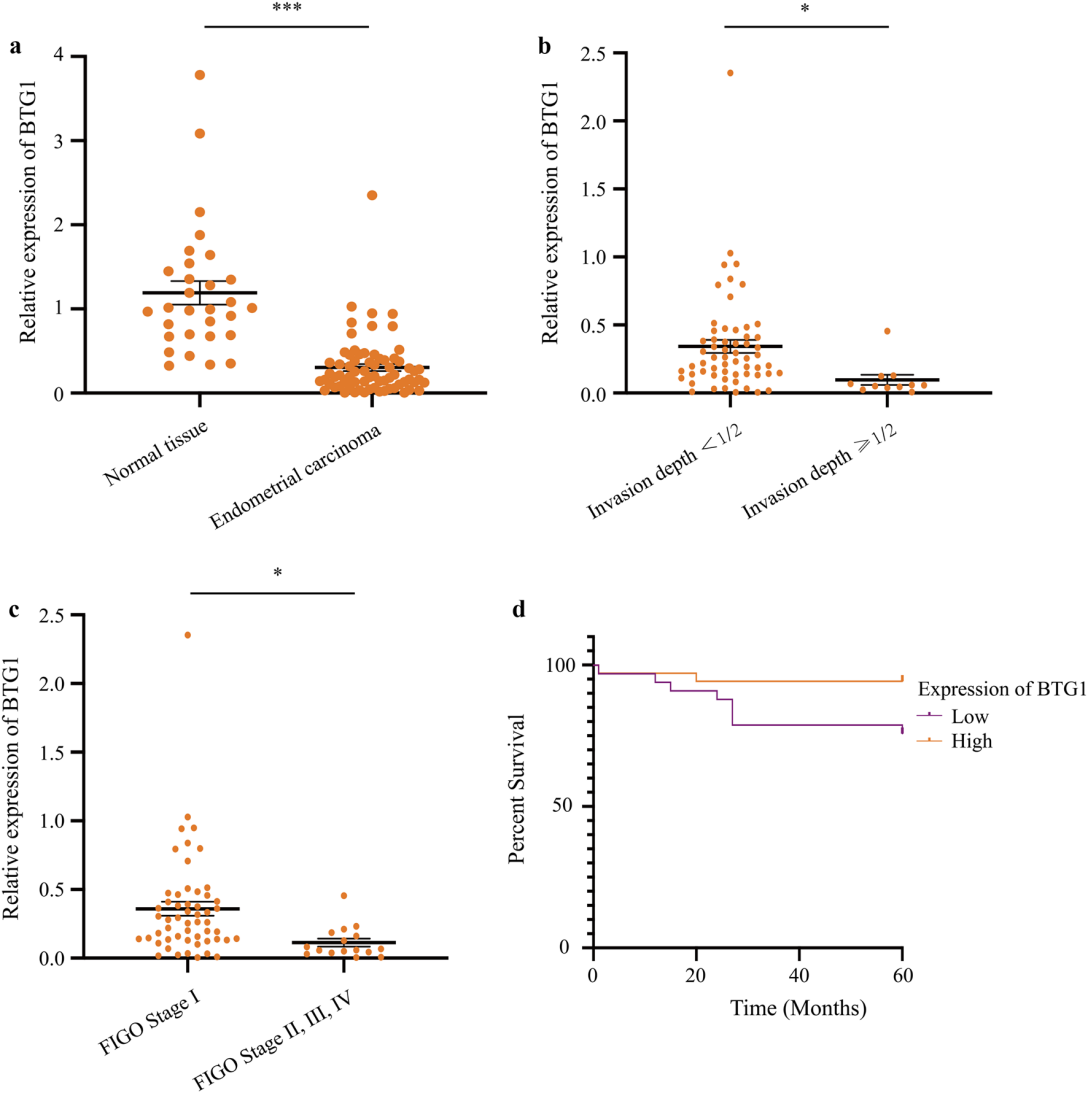
Low expression of BTG1 in EC was significantly correlated with OS shortening. **a** qRT-PCR detects the expression of BTG1 in EC samples and normal tissue samples. **b** Expression of BTG1 in EC based on invasion depth. **c** Expression of BTG1 in EC based on FIGO stage. **d** Effect of BTG1 expression level on EC patient survival. *P < 0.05,**P < 0.01 and ***P < 0.001

qRT-PCR detection showed that compared with normal human endometrial tissue, BTG1 had low expression in human EC tissue (P ≤ 0.001, Fig. 3a). Clinicopathological analysis showed the expression of BTG1 was related to invasion depth (P = 0.031) and FIGO stage (P = 0.012). The relative invasion depth of BTG1 in EC with invasion depth ≥ 1/2 showed less expression than EC with an invasion depth < 1/2 EC. BTG1 was relatively under-expressed in FIGO stage II, III and IV compared to FIGO stage I (Fig. 3b, c). BTG1 and EC pathological tissue type (P = 0.450), fertility history (P = 0.694), lymphatic metastasis (P = 0.148), menopause (P = 0.206), estrogen receptor (ER) (P = 0.930), progesterone receptor (PR) (P = 0.163), and age of diagnosis (P = 0.227) were not related (Additional file 4: Figure S2A–2G).

### BTG1 expression affects the prognosis of EC

A total of 70 patients with EC were followed up until February 15, 2019. The longest and shortest survival times were 60 months and 1 month, respectively. A total of 13 of 70 patients with EC died. Univariate results indicated that the average survival time of the BTG1 high-expression group was 57.2 months, and the average survival time of the BTG1 low-expression group was 48.9 months. Low expression of BTG1 was significantly correlated with OS shortening (P = 0.041) (Fig. 3d). These results are consistent with the results we obtained based on TCGA.

### Functional enrichment of BTG1 in EC patients

**Fig. 4:**
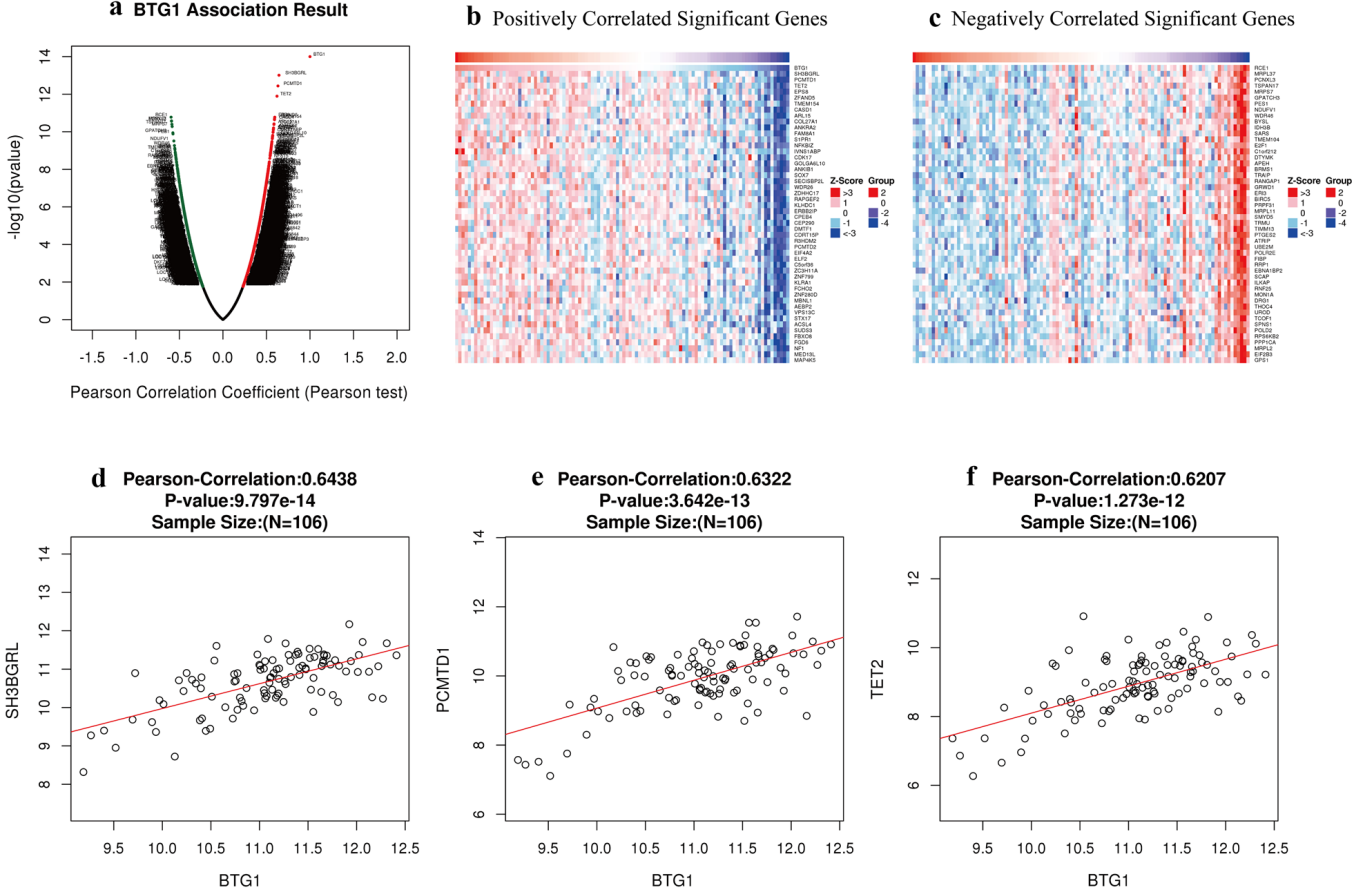
Genes related to BTG1 in LinkedOmics. **a** BTG1 association results. (volcano graph) **b** Positively correlated significant genes. (heat map) **c** Negatively correlated significant genes. (heat map) **d** Pearson-Correlation analyzes the correlation between BTG1 and SH3BGRL. **e** Pearson-Correlation analyzes the correlation between BTG1 and PCMTD1. **f** Pearson-Correlation analysis of the correlation between BTG1 and TET2

Using the functional module in LinkedOmics, the mRNA-related genes of 176 UCEC patients in TCGA were analyzed. As shown in the volcano graph, there were 2805 genes that significantly positively correlated with BTG1 (dark red dots), and 1267 genes that significantly negatively correlated with BTG1 (dark green dots) (FDR < 0.01) (Fig. 4a). The heat map showed that the top 50 gene sets were positively and negatively related to BTG1 (Fig. 4b, c). We queried the functions of the top 50 gene sets positively and negatively related to BTG1. The results showed that BTG1 plays an important role in regulating embryonic development, tumorigenesis, apoptosis, and cell cycle. The statistical scatter plot of each gene showed the expression of BTG1 and SH3BGRL (Pearson-Correlation: 0.6438, P = 9.797e−14), PCMTD1 (Pearson-Correlation: 0.6322, P = 3.642e−13), and TET2 (Pearson-Correlation: 0.6207, P = 1.273e−12) had a strong correlation (Fig. 4d, f).

**Fig. 5:**
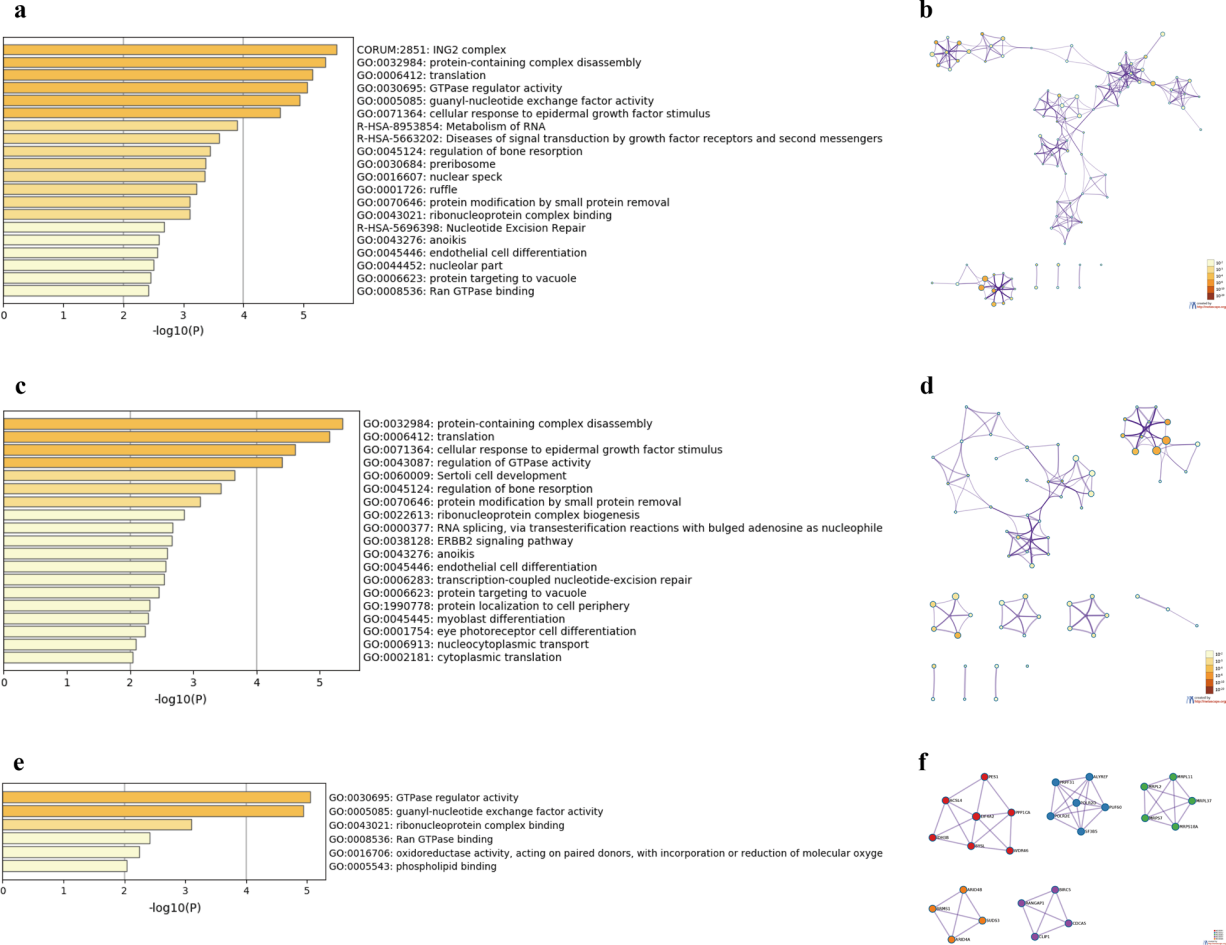
GO annotation of BTG1 related genes in EC. **a**, **b** The cellular components of genes related to BTG1 are enriched through bar graphs and networks. **c**, **d** Related biological processes related to BTG1 genes through bar graphs and networks. **e**, **f** The molecular functions of genes related to BTG1 are enriched through bar graphs and networks

**Fig. 6:**
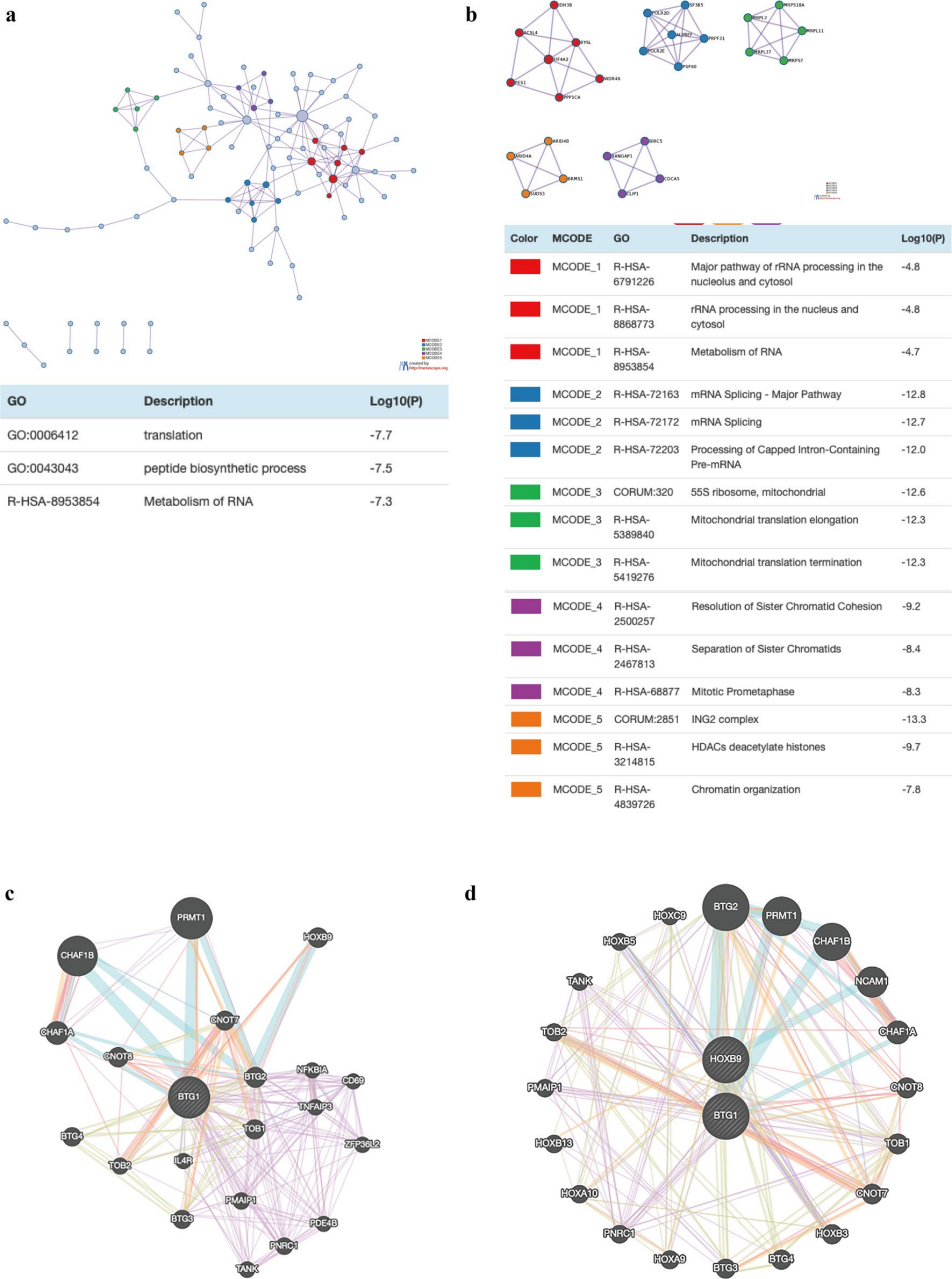
Protein–protein interaction (PPI) network. **a** PPI network in Metascape. **b** Independent feature richness analysis of five MCODE components in Metascape. **c** The protein interaction network constructed by GeneMANIA. **d** Mutual adjustment of BTG1 and HOXB9

Next, we used Metascape for GO enrichment analysis to analyze the function of BTG1 and the top 200 related genes. The results showed that BTG1 and its related genes are mainly involved in the ING2 complex, protein-containing complex disassembly, translation, GTPase regulator activity, exchange factor activity, and cellular response to epidermal growth factor stimulus (Fig. 5a, b, Additional file 5: Table S3). The biological processes of BTG1 and its related genes included protein-containing complex disassembly, translation, cellular response to epidermal growth factor stimulus, and regulation of GTPase activity (Fig. 5c, d, Additional file 6: Table S4). The molecular functions of BTG1 and its related genes included GTPase regulator activity and guanyl-nucleotide exchange factor activity (Fig. 5e, f, Additional file 7: Table S5). In order to further analyze the relationship between BTG1 and UCEC, we conducted a PPI network analysis and analysis of important genetic components in MCODE. Research suggests that its biological function includes the following aspects: major pathway of rRNA processing in the nucleolus and cytosol, rRNA processing in the nucleus and cytosol, metabolism of RNA, mRNA splicing, major pathway, mRNA splicing, processing of capped intron-containing pre-mRNA, 55S ribosome, mitochondrial, mitochondrial translation elongation, mitochondrial translation termination, resolution of sister chromatid cohesion, separation of sister chromatids, mitotic prometaphase, ING2 complex, HDACs deacetylate histones, and chromatin organization (Fig. 6a, b).

### BTG1 miRNA target and interacting protein network in EC

In order to further study the functional targets of BTG1 in EC, we used miRDB to obtain 58 miRNAs that can bind to BTG1 (Additional file 8: Table S6). Using the protein interaction network constructed by GeneMANIA (Fig. 6c), we obtained interacting proteins, including HOXB9, a transcription factor involved in cell proliferation and differentiation. We further revealed the mutual regulation of BTG1 and HOXB9 (Fig. 6d).

### BTG1 inhibits cell proliferation, migration, invasion, and promotes apoptosis in ECCs

**Fig. 7:**
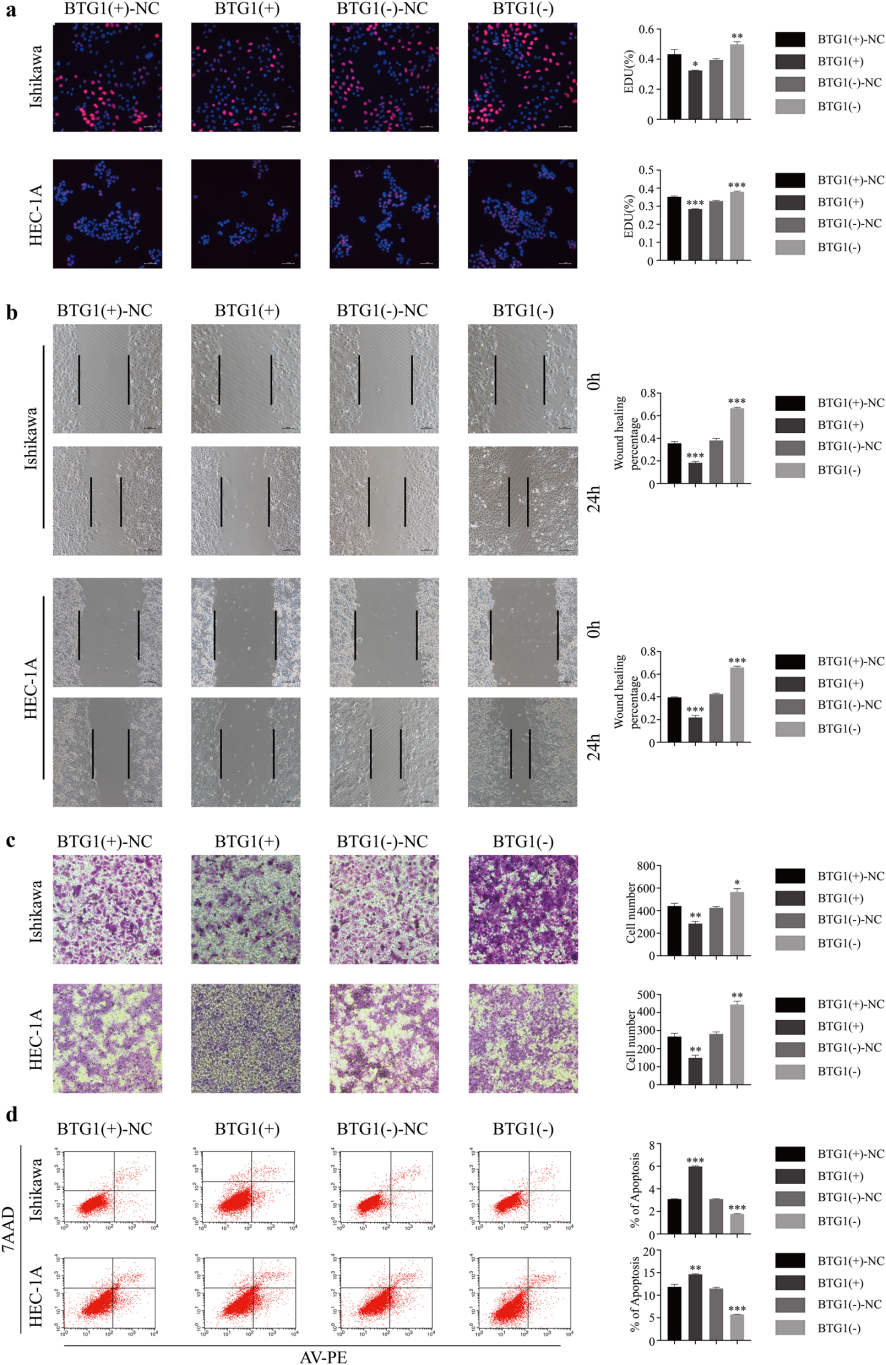
BTG1 inhibits the malignant biological behavior of ECC. **a** The EDU test was used to determine the effect of BTG1 on the proliferation of Ishikawa and HEC-1A cell lines. **b** Wound healing assay is used to determine the effect of BTG1 on the migration of Ishikawa and HEC-1A cell lines. **c** Transwell invasion assay to determine the effect of BTG1 on the invasion ability of Ishikawa and HEC-1A cell lines. **d** Cell apoptosis assay was used to analyze the effect of BTG1 on the apoptosis of Ishikawa and HEC-1A cell lines. The data are expressed as mean ± SEM (n = 3, each group). *P < 0.05, **P < 0.01 and ***P < 0.001

In order to study the effect of BTG1 on the malignant biological behavior of ECCs, we first transfected Ishikawa cells and HEC-1A cells with a BTG1 overexpression plasmid, a BTG1 knockdown plasmid, or a corresponding negative control. The transfection efficiency was verified by qRT-PCR. A knockdown plasmid with the best knockdown effect (BTG1-RNAi (6008-2) was selected for subsequent experiments (Additional file 9: Figure S3). Through EDU experiments, we found that overexpression of BTG1 could inhibit the proliferation of Ishikawa cells and HEC-1A cells, while knockdown of BTG1 could promote the proliferation of Ishikawa cells and HEC-1A cells (Fig. 7a). We obtained through a wound healing assay that overexpression of BTG1 inhibited the invasion of Ishikawa cells and HEC-1A cells. In contrast, knocking down BTG1 promoted the migration of Ishikawa cells and HEC-1A cells (Fig. 7b). The transwell cell invasion assay showed that BTG1 overexpression inhibited the invasion of Ishikawa cells and HEC-1A cells. In contrast, knocking down BTG1 expression promoted the invasion of Ishikawa cells and HEC-1A cells (Fig. 7c). Finally, we used flow cytometry to examine the effect of BTG1 knockdown and overexpression on apoptosis in Ishikawa cells and HEC-1A cells. The results showed that BTG1 overexpression promoted apoptosis and BTG1 knockdown suppressed apoptosis (Fig. 7d).

### BTG1 inhibits the EMT process in endometrial cancer cells

**Fig. 8:**
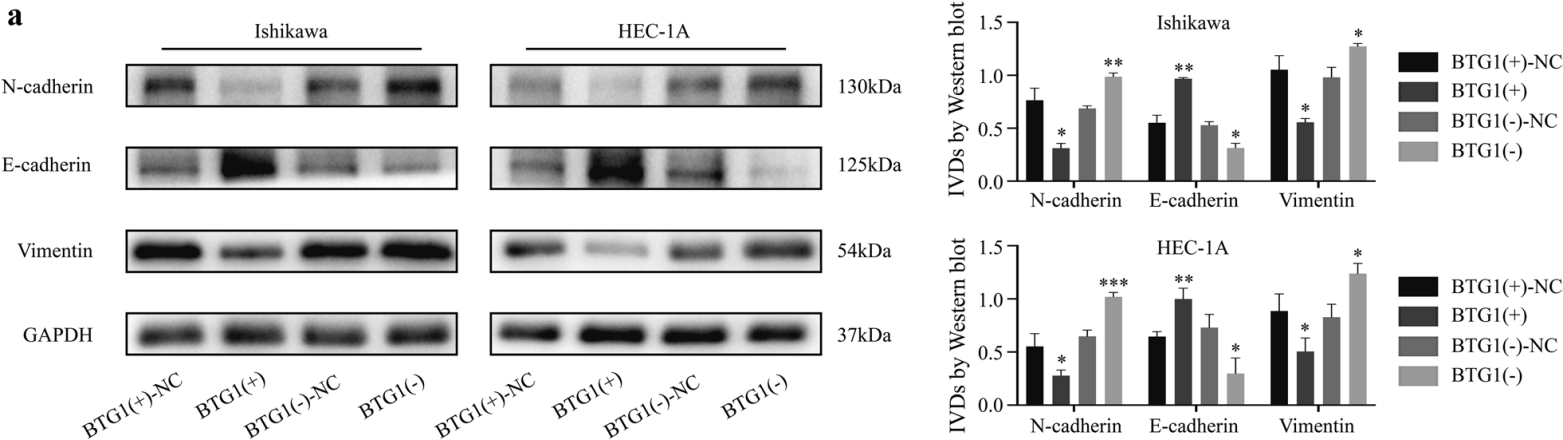
BTG1 suppresses the EMT process of ECC. **a** The effect of BTG1 on EMT related indicators N-cadherin, E-cadherin and Vimentin. The data are expressed as mean ± SEM (n = 3, each group). *P < 0.05, **P < 0.01 and ***P < 0.001

In order to explore the mechanism of BTG1 on the EMT process of ECCs, we detected the expression of E-cadherin, vimentin, and N-cadherin by western blot. Overexpression of BTG1 increased the expression of E-cadherin, while the expression of N-cadherin and vimentin decreased. Knocking down BTG1 reduced the expression of E-cadherin and increased the expression of N-cadherin and Vimentin (Fig. 8a). Our results indicate that BTG1 can inhibit the EMT process in ECCs.

## Discussion

EC is one of the three major malignant tumors of the female reproductive system [30]. The risk factors are related to excessive endometrial exposure to estrogen, age, obesity, hypertension, diabetes, and hereditary nonpolyposis colorectal cancer. The main treatment modality is still total hysterectomy with bilateral salpingo-oophorectomy. Radiation therapy and chemotherapy are also additional treatment options [31].

Abnormal expression of BTG1 in various tumors regulates their occurrence and development. The low expression of BTG1 in colon cancer is related to the clinicopathological characteristics, postoperative recurrence and survival of patients [32]. There is a low expression of BTG1 in gliomas, and it has a tumor suppressing effect in this case. Its anticancer effect is related to the inhibition of the Wnt/β-catenin pathway [33]. BTG1 downregulation is also seen in hepatocellular carcinoma and is significantly associated with disease-specific and relapse-free survival [34]. In gastric cancer, the downregulation of BTG1 leads to poor prognosis, especially in cases of proximal non-diffused and diffuse gastric cancer [35]. The expression of BTG1 is also low in breast cancer, and its overexpression has been associated with radiation therapy. Thus, by regulating the cell cycle and apoptosis-related signaling pathways, breast cancer treatment can be improved [36].

Using multiple databases, we analyzed the BTG1 expression in EC and studied its impact on the prognosis. In addition, we analyzed the expression of BTG1 in the functional regulation network. Based on the results of 546 UCEC cases in TCGA, the expression level of BTG1 mRNA in primary EC tissue was significantly lower than that in the normal control group (Fig. 2a). The expression of BTG1 is related to FIGO stage, weight, age, histological subtypes and TP-53 level (Fig. 2b–f). We then used the KM Plotter online analysis tool and the UALCAN database to predict that patients with high BTG1 expression had better prognosis (Fig. 2g, Additional file 3: Figure S1D). To further verify the expression of BTG1 in EC, we performed qRT-PCR experiments on 70 EC tissues and 30 normal endometrial tissues. We found that compared to normal human endometrial tissue, human EC tissue had lower BTG1 expression (Fig. 3a). Clinicopathological analysis suggests that the expression of BTG1 is related to the depth of invasion and FIGO stage (Fig. 3b, c). Expression of BTG1 was lower in EC with an infiltration depth ≥ 1/2. Expression of BTG1 was lower in those classified under FIGO stages II, III, and IV. However, BTG1 expression was not related to the EC pathological tissue type, fertility history, lymphatic metastasis, menopause, ER and PR status, and age of diagnosis (Additional file 4: Figure S2). We thus followed up these 70 EC patients. The longest and shortest survival times were 60 months and 1 month, respectively, and 13 out of 70 patients died. We concluded that the average survival time of the high BTG1 expression group was 57.2 months, and the average survival time of the low BTG1 expression group was 48.9 months. Low expression of BTG1 was significantly correlated with a shorter OS (Fig. 3d). Moreover, these results were consistent with the results we obtained based on TCGA. Next, we analyzed the gene sets positively and negatively related to BTG1 through Pearson-Correlation analysis. BTG1 was found to play an important role in regulating embryonic development, tumorigenesis, apoptosis, and the cell cycle (Fig. 4). The statistical scatter plot of each gene showed that the expressions of BTG1 and SH3BGRL (Pearson correlation: 0.6438, P = 9.797e−14), PCMTD1 (Pearson correlation: 0.6322, P = 3.642e−13), and TET2 (Pearson-Correlation: 0.6207, P = 1.273e−12) were strongly correlated. The scaffold protein SH3BGRL, observed, plays an important role in signal transduction, membrane transport, cytoskeleton rearrangement, and protein–protein interactions in other key cellular processes. The expression level of SH3BGRL is also considered a diagnostic index of breast cancer [37, 38]. PCMTD1 is used as a potential passenger gene in EC [39]. In addition, TET2 is a DNA methylcytosine dioxygenase that is closely related to tumor resistance and tumor metastasis [40–42]. Therefore, we speculate that BTG1 is involved in EC neutralization signal transduction, membrane transport, cytoskeleton rearrangement, EC drug resistance, and metastasis, to a large extent. The specific mechanism of action, however, requires further study. In addition, we conducted GO analysis of BTG1 and its related genes. BTG1 was related to protein-containing complex disassembly, translation, cellular response to epidermal growth factor stimulus, and regulation of GTPase activity (Fig. 5). We also predicted a miRNA and protein interaction network that binds to BTG1 (Additional file 8: Table S6). BTG1 and HOXB9 were found to be closely related (Fig. 6c, d). HOXB9 is a transcription factor of the HOX family and plays an important role in embryo development and tumor progression [43–46]. Studies have also shown that the expression of HOXB9 in EC is increased, and is related to histological grade and lymph node metastasis [47]. Therefore, the interaction between BTG1 and HOXB9 in EC and the underlying mechanism behind this need to be further researched.

BTG1 regulates the occurrence and development of various tumors. The low expression of BTG1 may be involved in the occurrence and development of pancreatic ductal adenocarcinoma [48]. miR-27a-3p regulates the proliferation and apoptosis of colon cancer cells by potentially targeting BTG1 [49]. lncRNA DGCR5 can be used as a ceRNA for BTG1 to inhibit the progression of gastric cancer [50]. However, there are no studies on EC. In order to study the effect of BTG1 on the malignant biological behavior of ECCs, we determined its effect on the proliferation, migration, and invasions in Ishikawa cells and HEC-1A cells through cell proliferation, wound healing, transwell invasion, and cell apoptosis assays. We found that BTG1 inhibited ECCs proliferation, migration, and invasion, and promote ECCs apoptosis (Fig. 7).

EMT is the process of obtaining mesenchymal features from epithelial cells. Most tumors undergo EMT during tumor progression. EMT promotes cell status to show stemness, invasiveness, drug-resistance, and the ability to metastasize to distant organs, thereby promoting cancer metastasis and recurrence [51]. The effect of BTG1 on EMT in EC is unclear. Therefore, we further studied the effect of BTG1 on EMT, and found that overexpression of BTG1 can inhibit the EMT process. In contrast, BTG1 knockdown promoted the EMT process (Fig. 8). However, the mechanism behind the regulatory effect of BTG1 on EMT still needs further study.

## Conclusion

In summary, based on the TCGA database, we utilized a variety of bioinformatics tools and databases to explore the expression and prognosis of BTG1. We used functional enrichment of BTG1 and related genes in EC patients through the bioinformatics website. We also analyzed the BTG1 miRNA targets and interacting protein networks. BTG1 played an important role in the occurrence and development of EC. BTG1, as a tumor suppressor gene, shows low expression in EC. BTG1 can inhibit the malignant biological behavior and EMT process of ECCs. In light of the above, we believe that BTG1 can be used as a potential prognostic biomarker for EC.

## Supplementary information

**Additional file 1: Table S1.** Primer Sequences.

**Additional file 2: Table S2.** Sequences of plasmid.

**Additional file 3: Figure S1.** A. The gene expression profile across all tumor samples and paired normal tissues in GEPIA. (Bar plot) B. Expression of BTG1 in UCEC based on patient’s race in UALCAN. C. Expression of BTG1 in UCEC based on Menopause status in UALCAN. D. Effect of BTG1 expression level on UCEC patient survival in UALCAN. E. Effect of BTG1 expression level & body weight on UCEC patient survival in UALCAN. F. Effect of BTG1 expression level & menopause status on UCEC patient survival in UALCAN. G. Effect of BTG1 expression level & Race on UCEC patient survival.

**Additional file 4: Figure S2.** A. Expression of BTG1 in EC based on pathological tissue type. B. Expression of BTG1 in EC based on fertility history. C. Expression of BTG1 in EC based on lymphatic metastasis. D. Expression of BTG1 in EC based on menopause. E. Expression of BTG1 in EC based on ER. F. Expression of BTG1 in EC based on PR. G. Expression of BTG1 in EC based on age.

**Additional file 5: Table S3.** Significantly enriched GO annotations (Cellular Components) of BTG1 in endometrial carcinoma in Metascape.

**Additional file 6: Table S4.** Significantly enriched GO annotations (Biological Processes) of BTG1 in endometrial carcinoma in Metascape.

**Additional file 7: Table S5.** Significantly enriched GO annotations (Molecular Functions) of BTG1 in endometrial carcinoma in Metascape.

**Additional file 8: Table S6.** The miRNAs binding to BTG1 in miRDB.

**Additional file 9: Figure S3.** A. Screening of BTG1 knockdown plasmid.

## Data Availability

All data generated or analyzed during this study are included in this published article.

## Abbreviations

EC: Endometrial carcinoma
BTG1: B-cell translocation gene 1
ATF4: Activating transcription factor 4
SCD1: Stearoyl-CoA desaturase-1
IECs: Intestinal epithelial cells
TCGA: The Cancer Genome Atlas
ECCs: Endometrial carcinoma cells
EMT: Epithelial-to-mesenchymal transition
FIGO: The International Federation of Gynecology and Obstetrics
ER: Estrogen receptor
PR: Progesterone receptor
KM: Kaplan–Meier
ANOVA: One-way analysis of variance
qRT-PCR: Quantitative real-time polymerase chain reaction
