# Supplementary material for: BTG1 inhibits malignancy as a novel prognosis signature in endometrial carcinoma
Source: Cancer Cell Int. 2020 Oct 7;20:490. doi: 10.1186/s12935-020-01591-3 (PMC7542768; doi:10.1186/s12935-020-01591-3)
Supplement: Supplementary file 1 — Additional file 1: Table S1. Primer Sequences. [file 12935_2020_1591_MOESM1_ESM.docx]

| Gene name | Primer Sequence (5’ to 3’) |
| --- | --- |
| BTG1 | Forward: TTACCGTTGTATTCGCATCAAC |
|  | Reverse: CCATCCTCTCCAATTCTGTAGG |
| GAPDH | Forward: GCACCGTCAAGGCTGAGAAC |
|  | Reverse: TGGTGAAGACGCCAGTGGA |

Table S1. Primer Sequences
