# Supplementary material for: BTG1 inhibits malignancy as a novel prognosis signature in endometrial carcinoma
Source: Cancer Cell Int. 2020 Oct 7;20:490. doi: 10.1186/s12935-020-01591-3 (PMC7542768; doi:10.1186/s12935-020-01591-3)
Supplement: Supplementary file 2 — Additional file 2: Table S2. Sequences of plasmid. [file 12935_2020_1591_MOESM2_ESM.docx]

| Name | Sequence |
| --- | --- |
| BTG1-RNAi (6007-1) | 5’- cgCCGTGTCCTTCATCTCCAA-3’ |
| BRG1-RNAi (6008-2) | 5’- gcAAATGGTAGACAGCCGAAT-3’ |
| BTG1-RNAi (6009-2) | 5’- TCCTACAGAATTGGAGAGGAT-3’ |
| CON036(BTG1-RNAi) | 5’-TTCTCCGAACGTGTCACGT-3’ |

Table S2. Sequences of plasmid
