# Supplementary material for: BTG1 inhibits malignancy as a novel prognosis signature in endometrial carcinoma
Source: Cancer Cell Int. 2020 Oct 7;20:490. doi: 10.1186/s12935-020-01591-3 (PMC7542768; doi:10.1186/s12935-020-01591-3)
Supplement: Supplementary file 5 — Additional file 5: Table S3. Significantly enriched GO annotations (Cellular Components) of BTG1 in endometrial carcinoma in Metascape. [file 12935_2020_1591_MOESM5_ESM.docx]

| GO | Category | Description | Count | % | Log10(P) | Log10(q) |
| --- | --- | --- | --- | --- | --- | --- |
| GO:0030684 | GO Cellular Components | preribosome | 5 | 2.54 | -3.37 | -0.56 |
| GO:0016607 | GO Cellular Components | nuclear speck | 11 | 5.58 | -3.36 | -0.56 |
| GO:0001726 | GO Cellular Components | ruffle | 7 | 3.55 | -3.21 | -0.56 |
| GO:0000313 | GO Cellular Components | organellar ribosome | 5 | 2.54 | -3.15 | -0.56 |
| GO:0044452 | GO Cellular Components | nucleolar part | 6 | 3.05 | -2.5 | -0.21 |
| GO:0005681 | GO Cellular Components | spliceosomal complex | 6 | 3.05 | -2.33 | -0.1 |
| GO:0005912 | GO Cellular Components | adherens junction | 11 | 5.58 | -2.28 | -0.1 |
| GO:0005667 | GO Cellular Components | transcription factor complex | 8 | 4.06 | -2.04 | 0 |
| GO:0032420 | GO Cellular Components | stereocilium | 3 | 1.52 | -2.02 | 0 |

Table S3. Significantly enriched GO annotations (Cellular Components) of BTG1 in endometrial carcinoma in Metascape
