# Supplementary material for: BTG1 inhibits malignancy as a novel prognosis signature in endometrial carcinoma
Source: Cancer Cell Int. 2020 Oct 7;20:490. doi: 10.1186/s12935-020-01591-3 (PMC7542768; doi:10.1186/s12935-020-01591-3)
Supplement: Supplementary file 6 — Additional file 6: Table S4. Significantly enriched GO annotations (Biological Processes) of BTG1 in endometrial carcinoma in Metascape. [file 12935_2020_1591_MOESM6_ESM.docx]

| GO | Category | Description | Count | % | Log10(P) | Log10(q) |
| --- | --- | --- | --- | --- | --- | --- |
| GO:0032984 | GO Biological Processes | protein-containing complex disassembly | 13 | 6.6 | -5.36 | -1.25 |
| GO:0006412 | GO Biological Processes | translation | 19 | 9.64 | -5.15 | -1.25 |
| GO:0071364 | GO Biological Processes | cellular response to epidermal growth factor stimulus | 5 | 2.54 | -4.61 | -1.01 |
| GO:0043087 | GO Biological Processes | regulation of GTPase activity | 14 | 7.11 | -4.4 | -0.98 |
| GO:0060009 | GO Biological Processes | Sertoli cell development | 3 | 1.52 | -3.66 | -0.5 |
| GO:0045124 | GO Biological Processes | regulation of bone resorption | 4 | 2.03 | -3.44 | -0.32 |
| GO:0070646 | GO Biological Processes | protein modification by small protein removal | 9 | 4.57 | -3.1 | -0.21 |
| GO:0022613 | GO Biological Processes | ribonucleoprotein complex biogenesis | 11 | 5.58 | -2.85 | -0.01 |
| GO:0000377 | GO Biological Processes | RNA splicing, via transesterification reactions with bulged adenosine as nucleophile | 9 | 4.57 | -2.67 | 0 |
| GO:0038128 | GO Biological Processes | ERBB2 signaling pathway | 3 | 1.52 | -2.66 | 0 |
| GO:0043276 | GO Biological Processes | anoikis | 3 | 1.52 | -2.59 | 0 |
| GO:0045446 | GO Biological Processes | endothelial cell differentiation | 5 | 2.54 | -2.56 | 0 |
| GO:0006283 | GO Biological Processes | transcription-coupled nucleotide-excision repair | 4 | 2.03 | -2.53 | 0 |
| GO:0006623 | GO Biological Processes | protein targeting to vacuole | 3 | 1.52 | -2.45 | 0 |
| GO:1990778 | GO Biological Processes | protein localization to cell periphery | 8 | 4.06 | -2.31 | 0 |
| GO:0045445 | GO Biological Processes | myoblast differentiation | 4 | 2.03 | -2.28 | 0 |
| GO:0001754 | GO Biological Processes | eye photoreceptor cell differentiation | 3 | 1.52 | -2.24 | 0 |
| GO:0006913 | GO Biological Processes | nucleocytoplasmic transport | 8 | 4.06 | -2.09 | 0 |
| GO:0002181 | GO Biological Processes | cytoplasmic translation | 4 | 2.03 | -2.04 | 0 |

Table S4. Significantly enriched GO annotations (Biological Processes) of BTG1 in endometrial carcinoma in Metascape
