# Supplementary material for: BTG1 inhibits malignancy as a novel prognosis signature in endometrial carcinoma
Source: Cancer Cell Int. 2020 Oct 7;20:490. doi: 10.1186/s12935-020-01591-3 (PMC7542768; doi:10.1186/s12935-020-01591-3)
Supplement: Supplementary file 7 — Additional file 7: Table S5. Significantly enriched GO annotations (Molecular Functions) of BTG1 in endometrial carcinoma in Metascape. [file 12935_2020_1591_MOESM7_ESM.docx]

| GO | Category | Description | Count | % | Log10(P) | Log10(q) |
| --- | --- | --- | --- | --- | --- | --- |
| GO:0030695 | GO Molecular Functions | GTPase regulator activity | 12 | 6.09 | -5.06 | -1.57 |
| GO:0005085 | GO Molecular Functions | guanyl-nucleotide exchange factor activity | 10 | 5.08 | -4.94 | -1.57 |
| GO:0043021 | GO Molecular Functions | ribonucleoprotein complex binding | 6 | 3.05 | -3.1 | -0.28 |
| GO:0008536 | GO Molecular Functions | Ran GTPase binding | 3 | 1.52 | -2.42 | 0 |
| GO:0016706 | GO Molecular Functions | oxidoreductase activity, acting on paired donors, with incorporation or reduction of molecular oxyge | 3 | 1.52 | -2.24 | 0 |
| GO:0005543 | GO Molecular Functions | phospholipid binding | 9 | 4.57 | -2.04 | 0 |

Table S5. Significantly enriched GO annotations (Molecular Functions) of BTG1 in endometrial carcinoma in Metascape
