# Supplementary material for: BTG1 inhibits malignancy as a novel prognosis signature in endometrial carcinoma
Source: Cancer Cell Int. 2020 Oct 7;20:490. doi: 10.1186/s12935-020-01591-3 (PMC7542768; doi:10.1186/s12935-020-01591-3)
Supplement: Supplementary file 8 — Additional file 8: Table S6. The miRNAs binding to BTG1 in miRDB. [file 12935_2020_1591_MOESM8_ESM.docx]

| Target Rank | Target Score | miRNA Name | Gene ID | Gene Symbol | Transcript Accession | Gene Description |
| --- | --- | --- | --- | --- | --- | --- |
| 1 | 98 | hsa-miR-513a-5p | 694 | BTG1 | NM_001731 | BTG anti-proliferation factor 1 |
| 2 | 97 | hsa-miR-548t-5p | 694 | BTG1 | NM_001731 | BTG anti-proliferation factor 1 |
| 3 | 93 | hsa-miR-302a-3p | 694 | BTG1 | NM_001731 | BTG anti-proliferation factor 1 |
| 4 | 93 | hsa-miR-302c-3p | 694 | BTG1 | NM_001731 | BTG anti-proliferation factor 1 |
| 5 | 93 | hsa-miR-580-3p | 694 | BTG1 | NM_001731 | BTG anti-proliferation factor 1 |
| 6 | 93 | hsa-miR-302d-3p | 694 | BTG1 | NM_001731 | BTG anti-proliferation factor 1 |
| 7 | 93 | hsa-miR-302b-3p | 694 | BTG1 | NM_001731 | BTG anti-proliferation factor 1 |
| 8 | 92 | hsa-miR-520c-3p | 694 | BTG1 | NM_001731 | BTG anti-proliferation factor 1 |
| 9 | 92 | hsa-miR-373-3p | 694 | BTG1 | NM_001731 | BTG anti-proliferation factor 1 |
| 10 | 92 | hsa-miR-372-3p | 694 | BTG1 | NM_001731 | BTG anti-proliferation factor 1 |
| 11 | 91 | hsa-miR-1297 | 694 | BTG1 | NM_001731 | BTG anti-proliferation factor 1 |
| 12 | 91 | hsa-miR-454-3p | 694 | BTG1 | NM_001731 | BTG anti-proliferation factor 1 |
| 13 | 91 | hsa-miR-509-3-5p | 694 | BTG1 | NM_001731 | BTG anti-proliferation factor 1 |
| 14 | 91 | hsa-miR-509-5p | 694 | BTG1 | NM_001731 | BTG anti-proliferation factor 1 |
| 15 | 91 | hsa-miR-493-5p | 694 | BTG1 | NM_001731 | BTG anti-proliferation factor 1 |
| 16 | 91 | hsa-miR-2355-5p | 694 | BTG1 | NM_001731 | BTG anti-proliferation factor 1 |
| 17 | 89 | hsa-miR-3192-5p | 694 | BTG1 | NM_001731 | BTG anti-proliferation factor 1 |
| 18 | 88 | hsa-miR-130b-3p | 694 | BTG1 | NM_001731 | BTG anti-proliferation factor 1 |
| 19 | 88 | hsa-miR-301b-3p | 694 | BTG1 | NM_001731 | BTG anti-proliferation factor 1 |
| 20 | 88 | hsa-miR-301a-3p | 694 | BTG1 | NM_001731 | BTG anti-proliferation factor 1 |
| 21 | 88 | hsa-miR-22-3p | 694 | BTG1 | NM_001731 | BTG anti-proliferation factor 1 |
| 22 | 88 | hsa-miR-130a-3p | 694 | BTG1 | NM_001731 | BTG anti-proliferation factor 1 |
| 23 | 86 | hsa-miR-19a-3p | 694 | BTG1 | NM_001731 | BTG anti-proliferation factor 1 |
| 24 | 86 | hsa-miR-19b-3p | 694 | BTG1 | NM_001731 | BTG anti-proliferation factor 1 |
| 25 | 86 | hsa-miR-27b-3p | 694 | BTG1 | NM_001731 | BTG anti-proliferation factor 1 |
| 26 | 86 | hsa-miR-27a-3p | 694 | BTG1 | NM_001731 | BTG anti-proliferation factor 1 |
| 27 | 83 | hsa-miR-145-5p | 694 | BTG1 | NM_001731 | BTG anti-proliferation factor 1 |
| 28 | 83 | hsa-miR-876-3p | 694 | BTG1 | NM_001731 | BTG anti-proliferation factor 1 |
| 29 | 81 | hsa-miR-26a-5p | 694 | BTG1 | NM_001731 | BTG anti-proliferation factor 1 |
| 30 | 81 | hsa-miR-26b-5p | 694 | BTG1 | NM_001731 | BTG anti-proliferation factor 1 |
| 31 | 80 | hsa-miR-92b-3p | 694 | BTG1 | NM_001731 | BTG anti-proliferation factor 1 |
| 32 | 80 | hsa-miR-92a-3p | 694 | BTG1 | NM_001731 | BTG anti-proliferation factor 1 |
| 33 | 80 | hsa-miR-32-5p | 694 | BTG1 | NM_001731 | BTG anti-proliferation factor 1 |
| 34 | 78 | hsa-miR-519b-3p | 694 | BTG1 | NM_001731 | BTG anti-proliferation factor 1 |
| 35 | 78 | hsa-miR-519a-3p | 694 | BTG1 | NM_001731 | BTG anti-proliferation factor 1 |
| 36 | 78 | hsa-miR-363-3p | 694 | BTG1 | NM_001731 | BTG anti-proliferation factor 1 |
| 37 | 78 | hsa-miR-367-3p | 694 | BTG1 | NM_001731 | BTG anti-proliferation factor 1 |
| 38 | 78 | hsa-miR-25-3p | 694 | BTG1 | NM_001731 | BTG anti-proliferation factor 1 |
| 39 | 77 | hsa-miR-668-3p | 694 | BTG1 | NM_001731 | BTG anti-proliferation factor 1 |
| 40 | 73 | hsa-miR-374c-5p | 694 | BTG1 | NM_001731 | BTG anti-proliferation factor 1 |
| 41 | 71 | hsa-miR-513a-3p | 694 | BTG1 | NM_001731 | BTG anti-proliferation factor 1 |
| 42 | 71 | hsa-miR-513c-3p | 694 | BTG1 | NM_001731 | BTG anti-proliferation factor 1 |
| 43 | 67 | hsa-miR-203a-3p | 694 | BTG1 | NM_001731 | BTG anti-proliferation factor 1 |
| 44 | 66 | hsa-miR-488-3p | 694 | BTG1 | NM_001731 | BTG anti-proliferation factor 1 |
| 45 | 66 | hsa-miR-576-5p | 694 | BTG1 | NM_001731 | BTG anti-proliferation factor 1 |
| 46 | 66 | hsa-miR-374a-5p | 694 | BTG1 | NM_001731 | BTG anti-proliferation factor 1 |
| 47 | 65 | hsa-miR-759 | 694 | BTG1 | NM_001731 | BTG anti-proliferation factor 1 |
| 48 | 65 | hsa-miR-183-5p | 694 | BTG1 | NM_001731 | BTG anti-proliferation factor 1 |
| 49 | 65 | hsa-miR-873-3p | 694 | BTG1 | NM_001731 | BTG anti-proliferation factor 1 |
| 50 | 64 | hsa-miR-655-3p | 694 | BTG1 | NM_001731 | BTG anti-proliferation factor 1 |
| 51 | 64 | hsa-miR-153-3p | 694 | BTG1 | NM_001731 | BTG anti-proliferation factor 1 |
| 52 | 63 | hsa-miR-320d | 694 | BTG1 | NM_001731 | BTG anti-proliferation factor 1 |
| 53 | 63 | hsa-miR-320b | 694 | BTG1 | NM_001731 | BTG anti-proliferation factor 1 |
| 54 | 63 | hsa-miR-374b-5p | 694 | BTG1 | NM_001731 | BTG anti-proliferation factor 1 |
| 55 | 63 | hsa-miR-1185-1-3p | 694 | BTG1 | NM_001731 | BTG anti-proliferation factor 1 |
| 56 | 63 | hsa-miR-1185-2-3p | 694 | BTG1 | NM_001731 | BTG anti-proliferation factor 1 |
| 57 | 63 | hsa-miR-320c | 694 | BTG1 | NM_001731 | BTG anti-proliferation factor 1 |
| 58 | 60 | hsa-miR-4524a-3p | 694 | BTG1 | NM_001731 | BTG anti-proliferation factor 1 |

Table S6. The miRNAs binding to BTG1 in miRDB
